# Prevalence of abnormal kidney function in a rural population of Benin and associated risk factors

**DOI:** 10.1186/s12882-021-02316-y

**Published:** 2021-03-31

**Authors:** Gwladys N. Gbaguidi, Corine Y. Houehanou, Salimanou A. Amidou, Jacques Vigan, Dismand S. Houinato, Philippe Lacroix

**Affiliations:** 1 Faculty of Health Sciences, Epidemiology Laboratory of Chronic and Neurologic Diseases, University of Abomey-Calavi, Cotonou, Benin; 2 INSERM, Univ. Limoges, CHU Limoges, IRD, U1094 Tropical Neuroepidemiology, Institute of Epidemiology and Tropical Neurology, GEIST, 87000 Limoges, France; 3 ENATSE, University of Parakou, Parakou, Benin; 4 Nephrology Unit, CNHU Cotonou, Cotonou, Benin; 5 Neurology Unit, CNHU Cotonou, Cotonou, Benin; 6 Department of Vascular Medicine, CHU Limoges, 87000 Limoges, France

**Keywords:** Abnormal kidney function, Prevalence, Associated factors, Rural area, Benin

## Abstract

**Background:**

The global burden of kidney disease has increased in recent years worldwide. Risk factors for kidney disease are common in Africa, but data on their prevalence are lacking. This study aims to determine the prevalence of abnormal kidney function and associated factors among participants included in the TAnve HEalth Study (TAHES) cohort in Benin.

**Methods:**

This was a cross-sectional study nested within the TAHES cohort. It was carried out in 2019, among TAHES participants aged 25 years and above, living in Tanvè and Dékanmè, two villages located in southwestern Benin. Data on risk factors were collected using the World Health Organization’s STEPS questionnaire. Anthropometric measurements and capillary creatinine measurements were performed. Abnormal kidney function was defined as a low glomerular filtration rate (< 60 mL/min/1.73 m2).

**Results:**

Creatinine was measured among 1360 out of the 1583 participants in the cohort in 2019. The median age was 39 [32–53]. The prevalence of abnormal kidney function was 16.10%; 95%CI = [14.15–18.05]. The results of the multivariate logistic regression showed that the probability of abnormal kidney function increased significantly with age (adjusted OR (aOR) = 2.75; 95%CI = [1.83–4.14]), female gender (aOR = 2; 95%CI = [1.37–2.91]), hypertension (aOR = 1.54; 95%CI = [1.12–2.13]), high body mass index (aOR = 1, 56; 95%CI = [1.12–2.17]) and hyperglycemia (aOR = 2.86; 95%CI = [1.68–4.88]).

**Conclusion:**

The prevalence of abnormal kidney function was high. These data should guide national authorities and help to raise community awareness of the benefits of early detection of this condition.

## Background

The pattern of disease emergence in Sub-Saharan Africa (SSA) is changing, leading to an increase in non-communicable diseases (NCDs) while infectious diseases already burden these countries [1]. The emergence of NCDs in SSA reflects complex sociodemographic transitions characterized by improved survival into adulthood with relative aging of populations, rapid urbanization, and changes in diet, levels of activity, and habits such as increased smoking and alcohol consumption [1, 2]. Chronic kidney disease (CKD) is the final common pathway for many infections and NCDs, and is an independent risk factor for death from cardiovascular causes [3]. There is growing concern about the increase in its incidence and prevalence in recent years in developed and developing countries [3, 4]. In 2016, the average prevalence of all stages of CKD was estimated at 13.4% worldwide [5]. According to a meta-analysis that included 21 studies performed in SSA, the prevalence of CKD was estimated at 13.9% in 2014 [6].

Patients with CKD have a high risk of progression to end-stage renal disease (ESRD). The end-stage of this disease requires dialysis or kidney transplantation [7]. Although more than 2 million people worldwide need chronic kidney replacement therapy, only a minority of patients who have elevated risks of developing ESRD receive medical care [8]. CKD is a major public health challenge because the majority of people with the disease are neither detected nor adequately treated early [9]. The costs of ESRD management are very high, if not prohibitive, for the majority of the population. CKD worsens the quality of life and often leads to death. Early management of CKD could have a socio-economic benefit to the population and a country. Besides, cardiovascular risk factors (CVRFs) such as hypertension and diabetes are associated with CKD and are significantly growing in low- and middle-income countries (LMICs). Indeed, a 162.5% increase in the burden of diabetes and cardiovascular disease (CVD) is projected in SSA by 2045 [10].

The paucity of reliable population-based data on the prevalence of CKD in SSA was highlighted in two recent systematic reviews of CKD, with weaknesses in study design, laboratory methods for creatinine measurement, and the absence of standardized criteria for the definition of CKD being cited as reasons [6, 11]. This could be an obstacle to the adoption of appropriate or adapted preventive measures. Early detection and intervention could prevent or reduce complications from kidney function alterations and could reduce the progression of kidney disease and the risk of CVD in the long term [12].

In Benin, STEPS survey conducted in 2015 showed the high prevalence’s of several CVRFs in the adult Beninese population aged 24 to 65 years. More than a quarter of people surveyed had High Blood Pressure (HBP), and 4 % had raised blood glucose. The prevalence of HBP was high in urban and rural areas [13]. Very few studies have explored the frequency of CKD and its associated factors in the general population of Benin. Exploring the relevance of the proteinuria / creatinuria ratio in the screening of chronic renal disease, a study conducted in southern Benin found an altered glomerular filtration rate among 13 and 7% of participants in rural and urban areas, respectively [14]. Under these conditions of increasing in CVD, it is important to assess kidney function as accurately as possible because renal disease has different clinical presentations and patients are often asymptomatic [15]. In the current study, using data from the Benin cohort of the TAnve HEalth Study (TAHES), we determined population-based prevalence estimates of abnormal kidney function and studied its associated factors in a rural population.

## Methods

### Study design and population

This was a cross-sectional study including TAHES participants. The TAHES project is an ongoing prospective cohort that started in February 2015 with an annual follow-up using a door-to-door approach. The study is conducted in the villages of Tanvè and Dékanmè located in the Agbangnizoun district, in the southwest of Benin. All people aged 25 years and over and living in the two villages were included. Participants unable to respond were excluded [16]. Participants who did not have a creatinine result for technical measurement difficulty were excluded.

### Data collection

Data were collected during a door-to-door survey conducted from 26 January to 17 February 2019 by seven previously trained investigative teams. The World Health Organization (WHO) STEPS questionnaire on NCD risk factors was used for the collection [17]. Socio-demographic and socio-economic information, personal and family cardiovascular medical history, lifestyle (physical activity, tobacco use, alcohol consumption, etc.), physical measurements (weight, height, waist circumference, hip circumference, blood pressure in both arms), and capillary blood glucose were collected. Capillary creatinine was collected in addition to the standard protocol.

### Creatinine measurement and abnormal kidney function definition

Fasting blood creatinine levels were assessed using a portable analyzer (StatSensor Xpress Creatinine, Nova Biomedical, Waltham, MA, United States) with single-use biosensors [18, 19]. To estimate the Glomerular Filtration Rate (eGFR), the Modification of Diet in Renal Disease (MDRD) study formula was used [20, 21]. The MDRD Study equation was expressed as follows: 175x (serum creatinine [(mg/dl)]) -1.154 X (age [(year)]) -0.203*0.742 (if female) * 1.21 (if black). Abnormal kidney function was defined as low eGFR (eGFR < 60 mL/min/1.73 m^2^) [24].

Participants were also classified in five stages by eGFR level as follows [22]:

- ○ Stage 1: eGFR ≥90 mL/min/1.73 m^2^
- ○ Stage 2: eGFR 60–89 mL/min/1.73 m^2^
- ○ Stage 3: eGFR 30–59 mL/min/1.73 m^2^
- ○ Stage 4: eGFR 15–29 mL/min/1.73 m^2^
- ○ Stage 5: eGFR < 15 mL/min/1.73 m^2^

### Sociodemographic data

Sociodemographic data included age, gender, education levels (illiterate, primary level, above primary level), marital status (in couple, single, widowed or divorced), occupation (independent farmer or informal, private or formal employee, unemployed), and the monthly income (< 68 $US, 68 $US -179 $US, ≥ 180 $US).

### Cardiovascular risk factor definitions

Risk factors were defined according to the WHO STEPS manual [23]. Blood pressure was measured in both arms, using an electronic device (OMRON M3), with adequate cuffs for normal and large arms. It was measured three times at 5-min intervals, in a seated position after a rest of at least 15 min. The average of the last two values was used to define blood pressure. HBP was defined as systolic and/or diastolic blood pressure ≥ 140/90 mmHg in one of the two arms, or by currently receiving medication for hypertension. Hyperglycemia was defined as a fasting capillary blood glucose value ≥6.1 mmol/L or currently receiving diabetes medication. The Body Mass Index (BMI) was calculated as weight in kilograms divided by the square of the height in meters. High BMI was defined as BMI ≥ 25 kg/m^2^.

### Statistical analysis

The Shapiro-Wilk test was used to assess whether the quantitative variables were distributed in a normal mode. If so, mean and standard deviation (SD) were used as summary statistics and compared between two groups using the Student’s test. If not, median and percentile were used and the Wilcoxon test performed for comparisons. Numbers and percentages were used for qualitative variables, and the chi-squared test was used for comparisons. A comparison of socio-demographic characteristics and CVRFs was conducted between participants with normal and abnormal kidney function in the study. The prevalence of abnormal kidney function has been estimated according to the MDRD equation. The different stages of eGFR have also been described. Univariate logistic regression models were performed to identify associations between kidney function and covariates. All variables that had *p*-values < 0.20 in the univariate logistic regression model were introduced simultaneously in the multivariate model. Crude and adjusted odds ratios (aOR) were estimated. In the sensitivity analysis, we estimated the prevalence of abnormal kidney function as well as the different stages of eGFR using the CKD-EPI formula. The threshold of significance was set at *p* <  0.05. The analysis was performed by using R software (Version 3.6.1). We used cross-sectional reporting guidelines (STROBE) for reporting this study [24].

## Results

### Population characteristics

**Fig. 1:**
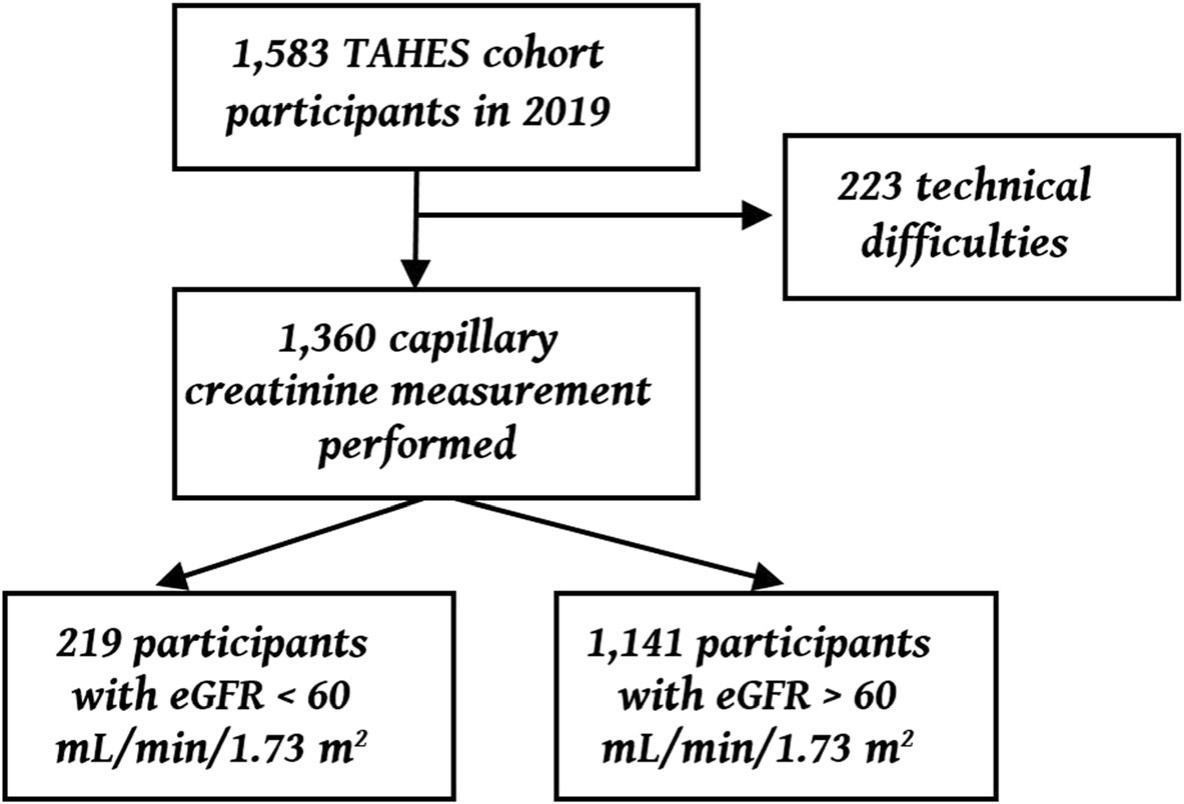
Flowchart of inclusion in this study population from the TAHES cohort, 2019

A total of 1583 participants were included in the TAHES cohort for the fourth annual follow-up survey in 2019. Among them, creatinine measurements were available for 1360 participants. There were 219 participants with eGFR deficiency (Fig. 1).

#### Sample characteristics

Table 1 presents the socio-demographic characteristics and the CVRFs comparison between participants with abnormal kidney function and those without. The median age of abnormal kidney function participants was 52 years, with an interquartile range of 38 to 66 years. The majority of abnormal kidney function participants were female. The prevalence of CVRFs in participants with abnormal kidney function was 47.5% for HBP, 37.4 for high BMI, and 11.9% for hyperglycemia.

**Table 1:** Socio-demographic characteristics and CVRFs for study participants according to the state of renal function, TAHES study, Benin 2019

	Abnormal kidney function (***n*** = 219)	Normal kidney function (***n*** = 1141)	***p***-value^*^
	Median [Q1-Q3] / n (%)	Median [Q1-Q3] / n (%)	
**Age (years)**	52 [38–66]	39 [31–50]	< 0.001
**Age (≥ 60 years)**	76 (34.7)	157 (13.8)	< 0.001
**Gender**			
Male	52 (23.7)	443 (38.8)	< 0.001
**Education levels**			
Illiterate	158 (72.1)	715 (62.7)	0.025
Primary level	52 (23.7)	353 (30.9)	
Above primary level	9 (4.1)	73 (6.4)	
**Marital status**			
In couple	153 (69.9)	980 (85.9)	< 0.001
Single, widowed or divorced	66 (30.1)	161 (14.1)	
**Occupation**			
Independent farmer or informal^a^	179 (81.7)	951 (83.3)	0.006
Private or formal employee	20 (9.1)	141 (12.4)	
Unemployed^b^	20 (9.1)	49 (4.3)	
**Monthly income ($US)**			
< 68	95 (43.4)	461 (40.4)	0.245
68–179	90 (41.1)	447 (39.2)	
≥ 180	34 (15.5)	233 (20.4)	
**HBP**	104 (47.5)	340 (29.8)	< 0.001
**High BMI**^c^	82 (37.4)	325 (28.5)	0.010
**Hyperglycemia**^d^ / **Diabetes Treatment**^e^	26 (11.9)	51 (4.5)	< 0.001

^*^ *p* value of the chi-squared test for qualitative variables and Student’s test for quantitative variables

^a^Independent farmer or informal (reseller, craftsman, motorcycle taxi, small independent with or without the Trade Register, laborer)

^b^Unemployed (student, apprentice, pensioner other)

^c^High BMI (BMI ≥ 25 kg/m2)

^d^Hyperglycemia capillary blood glucose ≥6.1 mmol / L

^e^*n* = 1140 for the normal kidney function group

### Prevalence of abnormal kidney function

According the MDRD definition, the prevalence of abnormal kidney function was estimated at 16.1%; (95% Confidence Interval (CI) = [14.1–18.1]) with 14.4, 1.6 and 0.1% for stages 3, 4 and 5, respectively (Table 2).

**Table 2:** Prevalence of abnormal kidney function and classification of eGFR according to the MDRD equation, TAHES Study, Benin 2019

eGFR by stages (eGFR in mL/min/1.73 m^**2**^)	***n*** = 1360	(%)
**Overall prevalence (eGFR < 60)**	**219**	**16.1 [14.1–18.1]**
**eGFR by stages**		
Stage 1 (eGFR ≥90)	452	33.2
Stage 2 (eGFR = 60–89)	689	50.7
Stage 3 (eGFR = 30–59)	196	14.4
Stage 4 (eGFR = 15–29)	22	1.6
Stage 5 (eGFR < 15)	1	0.1

### Associated factors

Table 3 presents the associations between CVRFs, sociodemographic characteristics, and kidney function status. In the univariate analysis, only monthly income was not associated with abnormal kidney function (*p* = 0.229). Considering people aged < 60 years as the reference group, the probability of having abnormal kidney function was significantly higher in the ≥60-year-old group (OR = 3.34; 95%CI = [2.41–4.63]; *p* <  0.001). Females were more at risk for abnormal kidney function (OR = 2.04; 95%CI = [1.46–2.84]; *p* <  0.001) than males. Primary school-level training was associated with a lower probability of abnormal kidney function (OR = 0.67; 95%CI = [0.48–0.93]; *p* = 0.022) compared to illiterate participants. Living alone increased the risk of abnormal kidney function (OR = 2.63; 95%CI = [1.88–3.66]; *p* <  0.001). The unemployed had significantly more risk for abnormal kidney function (OR = 2.17; 95%CI = [1.26–3.74]; *p* = 0.011) than self-employed farmers or people with informal occupations. Participants with HBP (OR = 2.13; 95%CI = [1.59–2.86]; *p* <  0.001), high BMI (OR = 1.50, 95%CI = [1.11–2.03]; *p* = 0.008) and those with hyperglycemia or under diabetes treatment (OR = 2.88, 95%CI = [1.75–4.73]; *p* <  0.001) respectively had significantly high risks of abnormal kidney function.

**Table 3:** Factors associated with abnormal kidney function, TAHES Study, Benin 2019

	Univariate analysis ***n*** = 1360		Multivariate analysis ***n*** = 1359	
	OR (95%CI)	***P*** value^*^	aOR (95%CI)	***P*** value^*^
Age (> 60 years)	3.34 (2.41,4.63)	< 0.001	2.57 (1.73,3.83)	< 0.001
**Gender**				
Male	1		1	
Female	2.04 (1.46,2.84)	< 0.001	1.98 (1.36,2.89)	< 0.001
**Level of education**		0.022		0.95
Illiterate	1		1	
Primary level	0.67 (0.48,0.93)		1.06 (0.72,1.54)	
Above primary level	0.56 (0.27,1.14)		1.09 (0.49,2.43)	
**Marital status**				
In couple	1			
Single, widowed or divorced	2.63 (1.88,3.66)	< 0.001	1.42 (0.95,2.13)	0.091
**Occupation**		0.011		0.278
Independent farmer or informal	1			
Private or formal employee	0.75 (0.46,1.24)		0.73 (0.43,1.26)	
Unemployed	2.17 (1.26,3.74)		1.36 (0.73,2.51)	
**Monthly income ($US)**		0.229		
< 68	1			
68–179	0.98 (0.71,1.34)			
≥ 180	0.71 (0.46,1.08)			
**HBP**	2.13 (1.59,2.86)	< 0.001	1.56 (1.13,2.15)	0.007
**High BMI**^**a**^	1.50 (1.11,2.03)	0.008	1.56 (1.12,2.17)	0.009
**Hyperglycemia**^**b**^ **/ diabetes treatment**	2.88 (1.75,4.73)	< 0.001	2.88 (1.68,4.92)	< 0.001

^*^p value of Wald test for binary variables or likelihood test for categorical variables with more than two modalities

^a^ High BMI (BMI ≥ 25 kg/m2)

^b^ Hyperglycemia capillary blood glucose ≥6.1 mmol/L

Results of the multivariable logistic regression showed that the probability of abnormal kidney function was significantly higher in the ≥60 year-old age group than in the < 60 year-old age group (aOR =2.75; 95%CI = [1.73–3.83]; *p* <  0.001). Women had a higher risk for abnormal kidney function than men (aOR = 1.98; 95%CI = [1.36–2.89]; *p* <  0.001). The probability of abnormal kidney function was significantly higher among participants with HBP (aOR = 1.56; 95%CI = [1.13–2.15]; *p* = 0.007), participants with high BMI (aOR = 1.56; 95%CI = [1.12–2.17]; *p* = 0.009) and in participants with hyperglycemia or diabetes (aOR = 2.88; 95%CI = [1.68–4.92]; *p* <  0.001) than among those without.

### Sensitivity analysis

By using the CKD-EPI equation, the estimated prevalence of abnormal kidney function was slightly lower (14.1%; CI 95% = [12.25–15.95]). This prevalence was 12.4, 1.7, and 0.1% for the moderate, severe, and terminal stages, respectively (Table 4).

**Table 4:** Prevalence of abnormal kidney function and classification of eGFR according to CKD Epi equation, TAHES Study, Benin 2019

eGFR by stages (eGFR in mL/min/1.73 m^**2**^)	***n*** = 1360	(%)
**Overall prevalence (eGFR < 60)**	**192**	**14.1 [12.25–15.95]**
**eGFR by stages**		
Stage 1 (eGFR ≥90)	567	41.7
Stage 2 (eGFR = 60–89)	601	44.2
Stage 3 (eGFR = 30–59)	168	12.4
Stage 4 (eGFR = 15–29)	23	1.7
Stage 5 (eGFR < 15)	1	0.1

## Discussion

Using low eGFR to define abnormal kidney function, we showed a prevalence of 16.1%. This could be explained by the large burden of CVRFs among our study participants (32.6% for HBP, 29.9% for high BMI, 5.7% for hyperglycemia). Our results are consistent with the worldwide range of fluctuations for CKD prevalence 8–16% [25]. However, this is higher than the CKD prevalence reported in previous studies conducted by Okwuonu et al. in Nigeria, Sumaili et al. in the Democratic Republic of Congo and Adeniyi et al. in South Africa, with prevalence rates of 7.8, 12.4, and 6.1%, respectively [4, 26, 27]. These low CKD prevalence rates could be explained by the population’s characteristics and differences in CKD measurements. Indeed, Okwuonu et al. in their study repeated the measurement of creatinine 3 months later. This certainly allowed them to obtain a better estimate of the prevalence of chronic kidney disease, unlike our study in which this measurement was only taken once. The South African study conducted by Matsha et al. found a CKD prevalence of 23.9% using the MDRD equation and 17.3% using the CKD-EPI in a mixed-ethnicity community in the city of Cape Town [28]. This estimation is higher than ours and could be explained by the fact that their study population was older (52.9 years old on average), had a high proportion of diabetics (26.4%) and smokers (40.5%).

We noted that the probability of abnormal kidney function increased significantly with age. Other studies have described such an association [29, 30]. This could be explained by the pathophysiological mechanisms, a decline in vital functions, and the increase in the prevalence of CVRFs with increasing age.

In our study, the risk of abnormal kidney function was also twice as high in women as in men. Similar observations were also made in several other studies [7, 31, 32]. This could be explained by our study population, which is more female, and by the average age of the participants with abnormal kidney function, which is relatively high in our study.

The difference in glomerular structure and hormone metabolism and the fact that females have less muscle mass than males are believed to play a major role in the differences in the prevalence of CKD between females and males [33, 34]. In contrast, some studies have shown that the incidence of CKD is lower in women than in men and that the decline in kidney function is slower in women than in men [29, 35, 36]. It has been shown that men consume more salt, phosphorus, and protein, and are more often obese and/or hypertensive [35]. These factors contribute to but do not fully explain the difference. Animal studies suggest that estrogens are nephroprotective and androgens are nephrotoxic. In humans, this subject has not yet been sufficiently studied and needs to be further investigated [34]. Adjusted for age and gender, CVRFs such as HBP, high BMI, and hyperglycemia or diabetes, were associated with a higher risk of abnormal kidney function. The relation between CKD and CVRFs such as hypertension and diabetes has been demonstrated in several studies [4, 30, 31]. Like other LMICs, the prevalence of hypertension is increasing in Benin [37]. According to the WHO STEPS survey conducted in Benin in 2015, the prevalence of hypertension was 26% with an almost identical distribution between rural and urban areas [38]. According to the previous study, in this cohort, 42% of hypertensive people were aware of their hypertensive condition and only 46.3% of them were treated [16]. This poor management of HBP could induce consequences for other organs, including the kidney. Concerning diabetes, in 2019, SSA had the lowest age-standardized global prevalence of diabetes in the world (4.7%). According to the International Diabetes Federation, however, this region has the highest proportion of undiagnosed diabetes cases (66.8%) [39]. Untreated or poorly treated, diabetes has serious consequences on several organs, including the kidneys. Hyperglycemia induces hyperfiltration and morphological alterations in the kidneys, which then leads to excessive excretion of albumin in the urine (albuminuria), damage to the podocytes, and loss of the surface area of the kidney [40, 41].

As the metabolic syndromes could be consequences of the combination of CVRFs, kidney dysfunction is also a real concern. The diagnosis and effective treatment of the early stages of kidney disease is especially important in Africa and other LMIC environments because the options for treating late complications such as ESRD are very limited or unavailable. This is in contrast to high-income countries where dialysis or transplantation is available to many patients who require these expensive therapies. Screening programs dedicated to individuals at higher risk of developing kidney disease (those with diabetes, with HBP) must be promoted.

### Strength and limits of the study

The prevalence of abnormal kidney function may be overestimated because creatinemia was measured only once. The delay of abnormality was not known and the persistence of the abnormality could not be established. A Moroccan study that repeated creatinine measurements showed that 32% of stage 3 CKD patients in the initial test did not have a low eGFR on retesting [42]. Using the single creatinine threshold to define a low eGFR could overdiagnose the prevalence of the disease. The cross-sectional design of our study does not allow us to establish the causal relationship between abnormal kidney function and CVRFs. Also, our study did not include urine protein/albumin measurements. These tests could help refine the diagnosis of renal dysfunction. As the TAHES cohort is still ongoing, in future follow-up surveys we plan to perform repeated creatinine tests and additional measurements for albumin in urine. These supplemental measures will contribute to improving the data on renal function impairment and will provide accurate diagnosis/information on the presence of CKD in the population. We also have a potential residual confusion bias. Indeed, the use of drugs (antibiotics, NSAIDs), the use of herbal medicine and the presence of systemic infections were not evaluated in this study. Therefore, these factors may have led to underestimating or overestimating the strength of association of certain factors such as hypertension. It would be relevant to consider these factors in further study. Some participants in TAHES were not included in the study due to technical difficulties during creatinine measurement. This could introduce potential selection bias in our study. The distribution of important risk factors such as hypertension and hyperglycemia, however, was not different between the participants included and those excluded for failure to measure creatinine. The single-point measurement of high blood pressure and hyperglycemia is also a limitation of our study as it could lead to an overestimation of their prevalence.

Despite these limitations, our study remains the largest study conducted in a large general population in SSA. This study has enabled us to estimate the prevalence of CKD using a rigorous, standardized evaluation methodology. This is the first study conducted in rural Benin on a large population with exhaustive recruitment of the study population. The assessment of capillary creatinine was carried out using a creatinine tester and strips. This could limit the potential measure bias.

## Conclusion

This study carried out on a large rural population in Benin shows that the prevalence of abnormal kidney function was high in this population. Abnormal kidney function is associated with the presence of HBP and blood glucose levels, disorders that are often unrecognized and rarely controlled in this population. These data support the development of interventions for the prevention and early detection of these risk factors. Repeating the creatinine measurements and taking into account the limitations of this study could provide a better estimate of the prevalence of chronic kidney disease in this population.

## Data Availability

The data that support the findings of this study are available from DSH and PL but restrictions apply to the availability of these data, which were used under license for the current study and so are not publicly available. Data are, however, available from the authors upon reasonable request and with permission from DSH and PL.

## Abbreviations

aOR: Adjusted Odd Ratios
BMI: Body Mass Index
CKD: Chronic Kidney Disease
CVD: Cardiovascular Disease
CVRFs: Cardiovascular Risk Factors
eGFR: Glomerular Filtration Rate
ESRD: End-Stage Renal Disease
HBP: High blood pressure
LMIC: Low-and Middle-Income Countries
MDRD: Modification of Diet in Renal Disease
NCD: Non-Communicable Diseases
SSA: Sub-Saharan Africa
SD: standard deviation
TAHES: TAnve HEalth Study
WHO: World Health Organization
